# Opioid-Induced Constipation: Pathophysiology, Clinical Consequences, and Management

**DOI:** 10.1155/2014/141737

**Published:** 2014-05-05

**Authors:** Lalit Kumar, Chris Barker, Anton Emmanuel

**Affiliations:** ^1^GI Physiology Unit, University College Hospital, 235 Euston Road, London NW1 2BU, UK; ^2^Pain Medicine, The Walton Centre for Neurology & Neurosurgery, Liverpool L9 7LJ, UK; ^3^Community Pain Service, Southport & Ormskirk NHS Trust, Town Lane, Kew, Southport PR8 6PN, UK

## Abstract

Although opioids offer potent analgesia for severe acute and chronic noncancer pain, adverse gastrointestinal effects potentially undermine their clinical utility. In particular, between 40% and 95% of patients develop opioid-induced constipation (OIC). Therefore, there is a consensus that patients should commence laxatives at the start of opioid therapy and continue throughout treatment. Nevertheless, laxatives are not routinely coprescribed with opioids. Even when concurrent laxatives are prescribed, approximately half the patients treated for OIC do not achieve the desired improvement. Moreover, laxatives do not target the underlying cause of OIC (opioid binding to the **μ**-receptors in the enteric system) and as such are not very effective at managing OIC. The failure of lifestyle modification and laxatives to treat adequately many cases of OIC led to the concurrent use of peripherally acting opioid antagonists (such as methylnaltrexone bromide and naloxone) to reduce the incidence of gastrointestinal adverse events without compromising analgesia. Judicious use of the various options to manage OIC should allow more patients to benefit from opioid analgesia. Therefore, this paper reviews the causes, consequences, and management of OIC to help clinicians optimise opioid analgesia.

## 1. Introduction

Opioids are increasingly used to alleviate severe acute and chronic noncancer pain, including back pain, spinal osteoarthritis, and failed back surgery. In recent years, opioid prescription has increased severalfold across Europe and USA [1, 2], leading to a rapid increase in fatalities in USA due to overdose and opioid abuse [2]. However, the National Institute for Health and Clinical Excellence (NICE) notes that opioids are underprescribed for severe pain due to concerns about addiction and side effects [3]. This paper reviews the causes, consequences, and management of “opioid-induced constipation” (OIC) to help clinicians, especially in primary care, use opioids effectively and safely.

## 2. Defining Constipation

Patients' and clinicians' definitions of constipation often differ. Many patients define constipation based on straining during defecation or stool consistency. However, functional constipation is best defined by the standard Rome III criteria: straining at stool; passage of lumpy or hard stools; sensation of incomplete evacuation or anorectal obstruction; the need to use manual manoeuvres to facilitate defecation; and passing fewer than three stools per week [4].

In addition, the Bowel Function Index (BFI) is a three-item questionnaire that assesses constipation based on ease of defecation, feeling of incomplete bowel evacuation, and the patients' judgement of constipation. The mean score is expressed on a scale between 0 and 100; the higher the score, the more severe the bowel dysfunction. A score of less than 28.8 represents normal bowel function, while changes of at least 12 points represent clinically meaningful differences [5]. The BFI is validated for OIC assessment [6].

## 3. Mechanisms Underlying OIC

Constipation can arise from the interaction of a plethora of underlying pathophysiologies, lifestyle factors, and medications [7]. While OIC, which is part of a broader constellation of symptoms called “opioid-induced bowel dysfunction” (OIBD), has been recognised for many years, health professionals still underestimate the condition's impact [7] on activities of daily living and quality of life (QoL). In addition, chronic constipation can result in haemorrhoid formation, rectal pain and burning, bowel obstruction, bowel rupture, and death [8], as well as upper gut dysfunctions, including gastrooesophageal reflux disease [9].

These gastrointestinal effects arise from opioid-mediated actions on the central nervous system (CNS) and gastrointestinal tract [8]. Centrally, opioids agonise four receptor subtypes: *μ*, *δ*, *κ*, and ORL-1 (opioid receptor-like-1). In addition to inducing analgesia, centrally acting opioids may reduce gastrointestinal propulsion, possibly by altering autonomic outflow from the CNS [8].

Nevertheless, the high density of *μ* receptors in the enteric system [9] appears to mediate most of opioid agonists' gastrointestinal effects [9], by reducing bowel tone and contractility, which prolongs transit time [10]. More frequent and stronger contractions of the circular muscles increase nonpropulsive contractions and, hence, enhance fluid absorption. In addition, reduced longitudinal muscle propulsive contractions exacerbate the trend to harder, drier stools. Opioid-mediated increased anal sphincter tone and decreased reflex relaxation in response to rectal distension contribute to the difficulty in rectal evacuation characteristic of OIC [10]. Studies of the human intestine suggest that *δ* and *κ* receptors make a lesser, but potentially clinically significant, contribution to opioid-induced inhibition of gastrointestinal muscle activity [9].

## 4. Clinical Consequences of OIC

Opioid agonism of CNS receptors can cause nausea, vomiting, sedation, respiratory depression, miosis, euphoria, and dysphoria. Opioid binding to peripheral receptors can result in hypotension, urinary retention, and OIC. Indeed, approximately 80% of patients taking opioids experience at least one side effect [11].

In contrast to most other opioid adverse events, tolerance does not typically develop to gastrointestinal side effects and these can potentially undermine the value of opioid analgesia. For example, constipation is one of the most common and bothersome adverse effects that comprise OIBD [12]. Estimates of the prevalence of OIC vary from 40% to 95% [13]. Nevertheless, the degree of distress and the duration of unpleasant symptoms show marked interpatient variation [14].

In addition, some patients taking opioids experience alternating constipation and diarrhoea, or diarrhoea alone, after several days without a bowel movement. These are cardinal signs of faecal impaction, which antidiarrhoeals or withdrawal of laxatives would exacerbate. Treating faecal impaction involves initial disimpaction, usually manual evacuation of faeces followed by an enema with either warm water and mineral oil or milk and molasses. Maintenance therapy should comprise regular polyethylene glycol (PEG), which is superior to lactulose at preventing recurrence [15].

Indeed, side effects limit patients' willingness to use opioids [13], thereby undermining clinical benefit. A systematic review of 11 studies encompassing 2877 patients with nonmalignant pain identified that opioid analgesia for more than six weeks significantly improved functional and QoL outcomes [16]. However, patients taking opioids for ≥6 months and suffering OIC were more likely to have time off work and feel impaired in their work and domestic performance (*P* < 0.05 for all comparisons) than those who did not develop OIBD [17]. Furthermore, the symptoms of OIC may be even more distressing for patients than the underlying chronic pain [18].

## 5. Management of OIC

**Figure 1:**
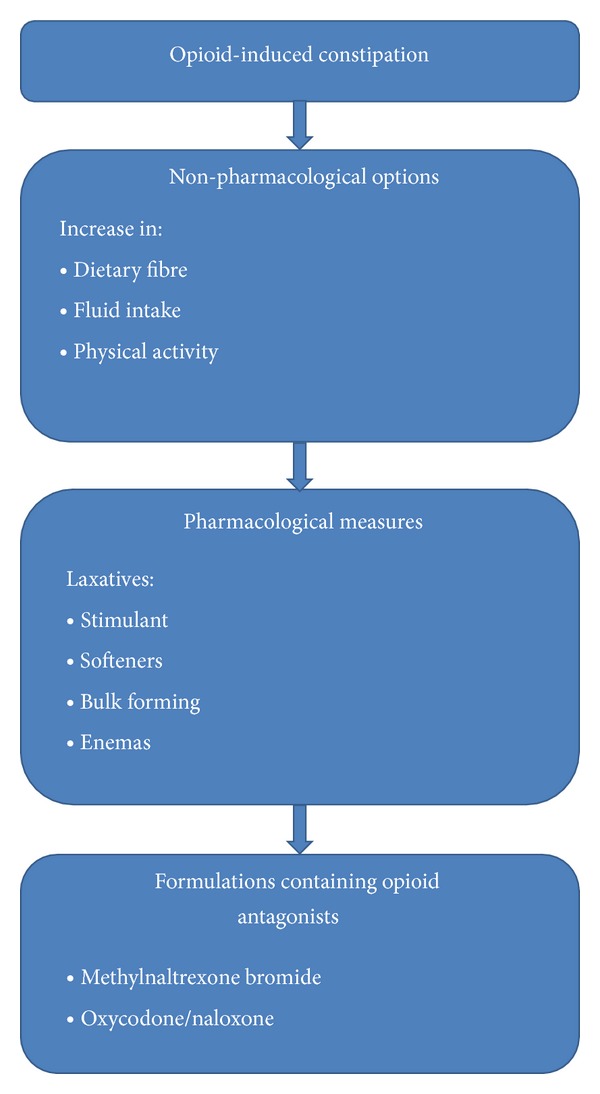
Treatment pathway for opioid-induced constipation.

OIC management encompasses either nonpharmacological or pharmacological approaches.  Figure 1 shows a management pathway for OIC.

### 5.1. Management of OIC with Nonpharmacological Options

Nonpharmacological management of OIC is based on lifestyle modification and should commence at the start of opioid therapy and continue for the duration of treatment. Typical measures include increasing consumption of dietary fibre, increasing oral fluids, and increasing physical activity [19].

However, nonpharmacological measures alone seldom successfully control OIC [19], most obviously because debilitated patients often experience difficulties complying with the regime. Chronic pain often compromises physical activity, for example. Furthermore, the efficacy of nonpharmacological interventions in the management of OIC remains unproven. Even patients with idiopathic constipation do not show differences in fibre intake compared with nonconstipated controls and increased fluid intake does not improve constipation unless the patient is initially dehydrated [20]. Nevertheless, nonpharmacological options should form a part of a general healthy lifestyle and can be combined with laxatives and other drugs for OIC.

### 5.2. Management of OIC with Laxatives

The commonest laxative regime for OIC combines a stimulant and stool softener. Gastrointestinal stimulants, such as senna or bisacodyl, increase muscle contractions mediated by an enteric reflex. Stool softeners act through one of three mechanisms.

- Surfactants, such as docusate, are emulsifiers that facilitate the admixture of fat and water in the faeces.
- Lubricants, such as mineral oil, delay absorption of water from stools in the colon, thus softening the faeces.
- Osmotics, such as lactulose or PEG, draws water into the colon, thereby hydrating the stools [21].

To the authors' knowledge, only one randomised controlled trial has assessed the efficacy and tolerability of PEG in OIC. In a randomised, double-blind, placebo-controlled study, PEG produced more “nonhard” stools in patients with methadone-induced constipation compared to placebo [22]. In patients with chronic constipation, two systematic reviews have suggested that PEG appears to be at least as effective as other laxatives [23, 24]. To the authors' knowledge, no treatment has been proven to be superior in head-to-head studies to PEG as a first-line intervention for chronic constipation or OIC.

Bulk-forming laxatives, such as psyllium (also called ispaghula), increase stool bulk, distend the colon, and stimulate peristalsis. However, bulk-forming laxatives are unsuitable for OIC. Opioids prevent peristalsis of the fibre-increased bulk, which exacerbates abdominal pain and, in some cases, contributes to bowel obstruction. Currently, there is a consensus that laxatives should commence at the start of opioid therapy and continue throughout treatment, although this is not routine. However, even when concurrent laxatives are prescribed, 54% of patients do not achieve the desired symptomatic improvement at least 50% of the time [13].

Laxatives are generally well tolerated. However, laxatives used for chronic constipation can cause side effects—including nausea, vomiting, diarrhoea, and abdominal pain—that might result in treatment cessation [25]. While uncommon, tolerance to stimulant laxatives can occur in patients with severe constipation and slow colonic transit [20]. Moreover, as laxatives do not address the mechanisms underlying OIC, many patients do not achieve adequate symptom relief [26]. Hence, there is the potential for supramaximal dosing with consequent unpredictability of effect. Alternative treatment strategies would, therefore, be advantageous. Combinations of opioid analgesics and peripheral antagonists might meet this need.

### 5.3. Management of OIC with an Opioid Antagonist

#### 5.3.1. Methylnaltrexone Bromide

Methylnaltrexone bromide, the first clinically available peripherally acting opioid antagonist, is indicated for OIC in patients receiving palliative care who showed an inadequate response to usual laxative therapy. Methylnaltrexone bromide, a selective antagonist at the *μ* receptor, poorly crosses the blood-brain barrier. As a result, methylnaltrexone bromide functions as an antagonist in the gastrointestinal tract, thereby decreasing opioids' constipating effects without undermining centrally mediated analgesia [19].

Phases I and II studies confirmed that methylnaltrexone bromide antagonised opioid-induced gastrointestinal effects, including decreasing gastric emptying time and increasing oral-caecal transit time. Moreover, a meta-analysis of 287 patients from palliative care settings enrolled in two randomised controlled studies calculated an odds ratio for the primary outcome of a rescue-free bowel movement within four hours versus placebo of 6.95 (95% CI: 3.83 to 12.61). The odds ratio of a rescue-free bowel movement within 24 hours was 5.42 (95% CI: 3.12 to 9.42) [25]. However, in one randomised clinical study, patients taking methylnaltrexone reported an increase in pain (3.4 ± 2.6) after 14 days, when asked to rate their current pain (on a scale of 0–10, with higher scores indicating greater severity) compared to those of placebo (2.7 ± 2.2). However, the authors did not report whether this reached statistical significance [27]. The difference observed in pain after 14 days between the placebo and methylnaltrexone groups was largely due to a reduction in pain within the placebo group.

Common adverse effects associated with methylnaltrexone bromide include abdominal pain, flatulence, nausea, dizziness, and diarrhoea. Methylnaltrexone bromide is contraindicated in known or suspected mechanical gastrointestinal obstruction [28]. Furthermore, patients with localized or diffuse reduction in the structural integrity of the gastrointestinal tract wall (e.g., associated with cancer, peptic ulcer, or Ogilvie's syndrome) have developed severe side effects, including gastrointestinal perforation, while taking methylnaltrexone bromide [28].

#### 5.3.2. Naloxone

An oral combination of oxycodone and naloxone—indicated to treat severe pain—might help prevent OIC. Naloxone shows a very low bioavailability (<2%) when given orally due to extensive hepatic metabolism. Therefore, oral naloxone binds at pharmacologically relevant concentrations only to peripheral opioid receptors in the gastrointestinal tract, which inhibits oxycodone's ability to modulate gastrointestinal function and, in turn, significantly reduces the risk of OIC [29, 30].

In a double-blind randomised control trial, the times to pain events (inadequate analgesia) were significantly shorter in the placebo group (i.e. placebo offered less pain control) compared with a combination of prolonged release (PR) oxycodone 3 PR/naloxone PR (*P* values between <0.0001 and 0.0003). Moreover, no statistically significant difference in time to pain event emerged between oxycodone PR/naloxone PR and oxycodone alone, confirming that naloxone PR does not undermine oxycodone's analgesic efficacy [30].

During a study comparing oxycodone PR/naloxone PR and oxycodone alone, investigators assessed patients using the BFI (see above) during six clinic visits over 12 weeks. BFI scores demonstrated a numerical reduction in the oxycodone PR/naloxone PR group compared to oxycodone alone at each visit, which reached statistical significance after one week (*P* < 0.004) and after four weeks (*P* < 0.005). The maximum difference in BFI score was recorded at four weeks: 45.4 for oxycodone alone and 26.1 with oxycodone PR/naloxone PR [29]. As mentioned above, a score under 28.8 represents normal bowel function and changes of at least 12 points are clinically meaningful [5]. Moreover, 30.2% of the oxycodone PR/naloxone PR group took laxatives compared to 54.4% of those receiving oxycodone PR (*P* < 0.0001) [29]. These benefits appear to be maintained in the long term. An open-label study with oxycodone PR/naloxone PR [31] found that analgesic efficacy was maintained for 12 months, while bowel function improved during the year-long study. Mean BFI score improved from 35.6 ± 27.74 at baseline to 20.4 ± 23.68 at 12 months.

The combination of oxycodone and naloxone is associated with a range of common adverse effects, including constipation, nausea, vomiting, diarrhoea, and abdominal pain. In two randomised controlled trials, there was a slightly higher incidence of adverse events in the oxycodone PR/naloxone PR groups compared to the oxycodone PR groups: 63.1% versus 52.6% and 55.8% versus 53.0%, respectively [30, 32]. Löwenstein and colleagues attributed the difference in adverse events to a higher incidence of abdominal pain in the oxycodone PR/naloxone PR group, possibly related to increased gut motility. In addition, more patients received oxycodone PR/naloxone PR (37.7%) than oxycodone PR (29.6%). Meissner and colleagues confirmed the suggestion that naloxone accounted for the increase in the incidence of adverse events [33]. By contrast, a separate randomised control study reported an equal incidence of adverse events in oxycodone PR/naloxone PR and oxycodone PR groups [29]. In general, however, adverse events associated with oxycodone plus naloxone tend to be mild to moderate and the incidence is often similar to placebo [29, 30]. Vondrackova and colleagues, for example, reported that 8.4% and 5.1% of the oxycodone PR/naloxone PR and placebo groups, respectively, developed constipation, while 6.5% and 7.0%, respectively, experienced nausea. The incidence of diarrhoea was 5.2% and 4.4%, respectively, in the oxycodone PR/naloxone PR and placebo groups [30]. Nevertheless, oxycodone/naloxone is contraindicated in patients with moderate to severe hepatic impairment and should be used cautiously in patients suffering from mild renal or mild hepatic impairment. Patients with severe renal impairment require careful monitoring. Oxycodone PR/naloxone PR is not recommended during pregnancy or lactation.

#### 5.3.3. Alvimopan

Alvimopan is a peripherally acting *μ*-opioid receptor antagonist (PAMORA) that does not cross the blood-brain barrier. These properties allow alvimopan to block the peripheral effects of opioids on the GI tract, without reversing centrally mediated analgesia.

The initial phase II trial results were encouraging and reported a dose-related increase in stool weight and in the incidence of effective bowel movements (ranging from 68% to 100% for different doses versus 30% for the placebo group). In addition, there was a decrease in the incidence of hard stools (ranging from 12% to 26% for alvimopan versus 67% for the placebo) and straining [34]. Similarly, a phase IIb trial reported a significant increase in the mean number of spontaneous bowel movements per week in the alvimopan group compared with the placebo group, +1.71 (95% CI 0.83–2.58) for alvimopan 0.5 mg BID, +1.64 (0.88–2.40) for alvimopan 1 mg QD, and +2.52 (1.40–3.64) for alvimopan 1 mg BID [35]. However, results are not consistent among all the studies. A phase III trial of 485 patients reported a nonsignificant increase in the proportion of patients experiencing spontaneous bowel movement in the alvimopan group (63%) compared to the placebo group (56%) [36]. Another study of patients with chronic cancer pain did not find any increase in the frequency of bowel movements with doses of 0.5 mg to 1 mg twice daily [37]. Consequent to these disappointing phase III data, further development of alvimopan to treat OIC was discontinued [38].

#### 5.3.4. Lubiprostone

Lubiprostone is a selective chloride channel-2 activator that acts locally in the small intestine leading to an increased fluid secretion and gut motility. Its efficacy in treating OIC has been assessed in two phase III trials [39]. The first trial reported a significant increase in spontaneous bowel movements at eight weeks and also overall for the entire 12-week study period. The mean number of spontaneous bowel movements per week increased from 1.42 to 4.54 with lubiprostone and from 1.46 to 3.81 with placebo [39]. However, the second trial did not show a significant increase in bowel movements in response to lubiprostone, according to the limited information available from the public website of the drug manufacturer [39, 40]. The limited data that exists for this drug led to the conclusion, by a recent meta-analysis, that more trials are required before a definitive recommendation can be made on the use of lubiprostone in OIC [41].

## 6. Conclusion

Despite alleviating severe acute and chronic noncancer pain, opioids are underprescribed due to concerns about addiction and side effects [3]. Indeed, 80% of patients taking opioids experience at least one side effect [11] including OIC, although the degree of distress shows marked variation [14]. However, in some cases, people with severe OIBD limit use of or discontinue opioids, to alleviate the additional pain and discomfort associated with OIBD [13].

OIC management can encompass nonpharmacological and pharmacological approaches. However, nonpharmacological measures alone seldom successfully control OIC symptoms but can be combined with pharmacological options [19]. Currently, there is a consensus that laxative treatment should commence with the opioid therapy and continue throughout treatment, although this is not routine. Even when concurrent laxatives are prescribed, approximately half of patients treated for OIC do not achieve the desired improvement [13]. Moreover, laxatives do not target the underlying cause of OIC-opioid binding to the *μ* receptors in the enteric system and as such are not very effective at managing OIC.

The failure of lifestyle modification and aggressive laxative therapy to treat OIC symptoms led to the development of analgesic formulations that include peripherally acting opioid antagonists. Judicious use of the various options to manage OIC should allow more patients with severe noncancer pain to benefit from opioid analgesia.

Although the safety and efficacy of opioid antagonists have been proven in several studies, none of the previous studies has established an improvement in quality of life with increased passage of bowel movements. This is a significant deficiency that needs to be addressed in the future studies.
